# From “negative” trial to positive clinical impact: mitigating eligibility criteria–induced temporal selection bias in emulated clinical trials

**DOI:** 10.1038/s44401-026-00082-3

**Published:** 2026-05-27

**Authors:** Xiaodi Li, Sivaraman Rajaganapathy, Xinyue Hu, MunHwan Lee, Jingna Feng, Jianfu Li, Yue Yu, Phil Fiero, Soulmaz Boroumand, Richard Larsen, Jun Chen, Pengyang Li, Jose James, Xiaoke Liu, Cui Tao, Nansu Zong

**Affiliations:** 1 Department of Artificial Intelligence and Informatics, Mayo Clinic, Rochester, MN USA; 2 Mayo Clinic Platform, Mayo Clinic, Rochester, MN USA; 3 Division of Computational Biology, Department of Quantitative Health Sciences, Mayo Clinic, Rochester, MN USA; 4 Division of Cardiology, Pauley Heart Center, Virginia Commonwealth University, Richmond, VA USA; 5 Mayo Clinic School of Graduate Medical Education, Rochester, MN USA; 6 MCHS Cardiology, Mayo Clinic, Rochester, MN USA

**Keywords:** Cardiology, Diseases, Health care, Medical research

## Abstract

While randomized controlled trials (RCTs) guide clinical practice, their completion may also influence real-world treatment patterns. We investigated whether the outcomes of trial emulation differ before and after the publication of the WARCEF (Warfarin versus Aspirin in Reduced Cardiac Ejection Fraction) randomized controlled trial. We emulated the WARCEF trial using EHR data from the Mayo Clinic Platform, comparing Warfarin and Aspirin in patients with heart failure and reduced ejection fraction (HFrEF). Analyses were stratified by the WARCEF completion date (July 2014), using intention-to-treat (ITT) frameworks. For the ITT analysis, the cohort size before 2014 consisted of 37,225 patients on Aspirin and 2022 on Warfarin, while after 2014, the cohort included 26,192 patients on Aspirin and 1080 on Warfarin. No significant treatment difference was observed before July 2014, 1.025 (95% CI: 0.6144 – 1.709, p = 0.9255), consistent with the findings of the WARCEF trial. However, after trial completion, Warfarin was associated with increased risk, 2.181 (95% CI: 1.676 – 2.839, p < 0.001). These findings initially suggested that the completion of the WARCEF trial and subsequent guideline updates were associated with changes in observed treatment effects in real-world emulation. However, further analyses indicate that the observed differences are primarily driven by the application of specific eligibility criteria rather than the trial completion date itself. In particular, the inclusion of restrictive eligibility criteria alters cohort composition and event timing, introducing selection bias that materially affects treatment effect estimates. This highlights that, in trial emulation studies, eligibility criteria can have a greater impact on observed outcomes than temporal stratification alone. Accurate emulation therefore requires careful alignment of eligibility definitions with the original trial design, as inappropriate or overly restrictive criteria may induce selection bias and distort causal inference, independent of trial publication timing.

## Introduction

Randomized controlled trials (RCTs) are widely regarded as the gold standard for establishing causal relationships in clinical research due to their ability to minimize bias and confounding^1^. Positive RCT findings often lead to updates in clinical guidelines and changes in medical practice^2,3^. However, the impact of negative or inconclusive RCTs on clinical practice is less clear. Studies have shown that while successful trials can rapidly shift treatment standards, negative results do not consistently lead to practice changes, and ineffective interventions may persist despite emerging evidence^4,5^. Our study uses real-world data while strictly adhering to the original trial criteria and time frame and reveals clear differences between data collected before and after the trial’s completion date.

The Warfarin versus Aspirin in Reduced Cardiac Ejection Fraction (WARCEF) trial was designed to determine whether Warfarin or Aspirin is more effective in preventing death and ischemic stroke among patients with heart failure and reduced ejection fraction (EF ≤ 35%). Although Warfarin has shown benefits in ischemic heart disease and atrial fibrillation, its role in heart failure patients without atrial fibrillation remained unclear. In this randomized, double-blind, multicenter trial enrolling 2,305 patients, WARCEF found no significant difference between Warfarin and Aspirin in time-to-death outcomes (hazard ratio [HR] 1.01; 95% CI, 0.85 to 1.20; p = 0.91). These findings are consistent with results from retrospective analyses^6–8^ and other randomized trials^9–11^, which similarly showed no significant reduction in thromboembolic events with Warfarin in heart failure patients.

Clinical guidelines evolve with emerging trial evidence, influencing both practice and future research. Prior to the WARCEF trial, recommendations on anticoagulation in heart failure with reduced ejection fraction (HFrEF) and sinus rhythm were largely extrapolated from atrial fibrillation populations, as reflected in the 2010 ESC Guidelines^12^. WARCEF addressed this gap by comparing Warfarin and Aspirin in HFrEF patients without atrial fibrillation, ultimately reporting no overall benefit due to offsetting reductions in ischemic stroke and increases in major bleeding. Following its publication, updated guidelines, including the 2020^13^ and 2024 ESC^14^ and 2022 AHA/ACC/HFSA^15^, recommended against routine anticoagulation in this population unless clear indications exist. Together with findings from the COMMANDER HF trial, WARCEF reshaped guideline recommendations and indirectly influenced cohort definitions, treatment comparisons, and outcome expectations in subsequent trial emulations.

This study seeks to emulate the WARCEF trial using real-world observational data from the Mayo Clinic Platform (MCP) to evaluate the comparative effectiveness of Warfarin versus Aspirin in patients with HFrEF. While our initial analyses stratified patients by the WARCEF trial completion date, further investigation revealed that differences in observed treatment effects are primarily driven by the application of specific eligibility criteria rather than temporal changes in clinical practice alone. In particular, restrictive inclusion criteria substantially alter cohort composition and event timing, introducing selection bias that can materially influence estimated treatment effects. These findings underscore the importance of carefully evaluating how individual eligibility criteria affect real-world trial emulation and highlight that eligibility-driven selection bias may outweigh temporal considerations when interpreting emulation results.

This study makes three contributions. (1) Using WARCEF as a case study, we show that apparent differences in treatment effects observed before and after trial completion may arise not from temporal changes in treatment effectiveness, but from the operationalization of eligibility criteria in real-world data, which can induce selection bias and distort comparative effectiveness estimates. (2) We demonstrate the feasibility of reproducible intention-to-treat trial emulation using large-scale EHR data from the Mayo Clinic Platform in patients with heart failure and reduced ejection fraction. (3) We provide methodological guidance on translating randomized trial protocols into observational settings, highlighting how eligibility definitions, particularly those with time-dependent availability, can have a greater impact on emulation results than temporal stratification alone.

## Results

### Study details

Table 1 summarizes the WARCEF trial, and the corresponding patient cohort identified from the MCP, including patient counts across different stratification methods and time periods. We conducted analyses using ITT (Intention-To-Treat) approaches and further divided the cohort into two groups: before and after July 2014.

**Table 1:** Study summaries and cohort characteristics for ITT analysis

Characteristics	MCP Data (Before July 2014)	MCP Data (After July 2014)	Original Study Data (Overall)
Medication	37,225 (Aspirin, 94.85%) | 2022 (Warfarin, 5.15%)	26,192 (Aspirin, 96.04%) | 1080 (Warfarin, 3.96%)	1163 (Aspirin, 50.46%) | 1142 (Warfarin, 49.54%)
Age	64.51 ± 13.05	63.74 ± 12.67	61 ± 11.3
Gender	21,937 (Male, 55.89%) | 17,310 (Female, 44.11%)	15,843 (Male, 58.09%) | 11,429 (Female, 41.91%)	1840 (Male, 79.83%) | 460 (Female, 19.96%) | <11 (Unknown, 0.22%)
Race	35,997 (White, 91.72%) | 1236 (Black, 3.15%) | 1202 (Other, 3.06%) | 812 (Unknown, 2.07%)	24,064 (White, 88.24%) | 1589 (Black, 5.83%) | 1145 (Other, 4.20%) | 474 (Unknown, 1.74%)	N/A
Eisenmenger’s syndrome	571 (1.45%)	139 (0.51%)	N/A
Decompensated heart failure	5876 (14.97%)	1850 (6.78%)	N/A
Heparin use	38,683 (98.06%)	26,748 (97.51%)	N/A
Dementia	3237 (8.25%)	1033 (3.79%)	N/A
Severe liver impairment	21,060 (53.66%)	13,546 (49.67%)	N/A
Severe uncontrolled hypertension	800 (2.04%)	327 (1.20%)	N/A
Creatinine	30,600 (77.97%)	18,376 (67.38%)	N/A
ACE inhibitor use	37,544 (95.66%)	25,951 (95.16%)	N/A
Recent stroke	1629 (4.15%)	495 (1.82%)	N/A
Ejection Fraction	55.08 ± 16.01	54.43 ± 16.17	N/A
Hypertension	19,555 (49.57%)	11,493 (41.90%)	N/A
Hyperlipidemia	28,057 (71.12%)	11,471 (41.82%)	N/A
Diabetes	12,628 (32.01%)	4,699 (17.13%)	N/A
Coronary artery disease	21,047 (53.35%)	7213 (26.30%)	N/A

Throughout the process, we devoted substantial effort to variable selection. In collaboration with physicians, we carefully reviewed and discussed which variables to include or exclude, guided by the official inclusion and exclusion criteria outlined on the trial’s website. We strictly included variables deemed essential and excluded those considered clear disqualifiers, such as chronic or paroxysmal atrial fibrillation (AF), presence of a mechanical valve, and age under 18. Remaining variables were treated as potential confounders. To closely emulate the WARCEF trial environment, we divided our dataset into two periods: before July 2014 and after July 2014. The pre-July 2014 cohort corresponds to the original trial period, capturing patients before the trial results were disseminated. The post-July 2014 cohort includes patients after the trial’s completion, reflecting the potential influence of WARCEF findings on real-world clinical practice.

### Detailed results

We assessed the mortality outcomes by comparing real-world emulated cohorts before and after the completion of the WARCEF trial in July 2014. Hazard ratio analyses, including key clinical risk factors, were conducted across the full study population. We excluded the Modified Rankin Score criterion because the proportion of patients outside the eligible range was very small (0.02%), and thus unlikely to materially influence the results. Moreover, inclusion of the Modified Rankin Score introduces selection bias by delaying effective cohort entry, such that the earliest observed death occurs in 2017. As shown in Table 2, before July 2014, the comparison between Warfarin and Aspirin showed no statistically significant difference in mortality. The hazard ratios were 1.025 (95% CI: 0.6144 – 1.709, p = 0.9255), suggesting a trend toward higher mortality with Warfarin but without reaching conventional significance. For the analysis before July 2014, significant risk factors included Age (Over 65) (HR = 2.894, p < 0.001), Smoking (Heavy) (HR = 2.613, p = 0.0084), Alcohol (Yes) (HR = 0.470, p = 0.0063), and Low molecular weight heparin (On medication) (HR = 1.663, p = 0.0296).

**Table 2:** Hazard Ratio for Mortality

ITT Before July 2014			ITT After July 2014		
Medication (Warfarin)			Medication (Warfarin)		
Hazard Ratio	*p*-value		Hazard Ratio	*p*-value	
1.025 (0.6144–1.709)	0.9255		2.181 (1.676–2.839)	<0.001	

Risk Factors			Risk Factors		
RF	Hazard Ratio	*p*-value	RF	Hazard Ratio	*p*-value
Age (Over 65)	2.894 (1.844–4.543)	<0.001	Age (Over 65)	1.872 (1.412–2.481)	<0.001
Smoking (Heavy)	2.613 (1.279–5.337)	0.0084	Smoking (Heavy)	2.036 (1.340–3.095)	<0.001
Alcohol (Yes)	0.470 (0.273–0.808)	0.0063	Alcohol (Yes)	0.473 (0.324–0.691)	<0.001
Low molecular weight heparin (On medication)	1.663 (1.052–2.630)	0.0296	Contraindications (Present)	0.521 (0.309–0.879)	0.0144
			Low molecular weight heparin (On medication)	1.585 (1.171–2.146)	0.0029
			NSAID (Present)	0.671 (0.471–0.958)	0.0278
			ACE inhibitor use (Present)	0.471 (0.270–0.821)	0.0079

In contrast, as shown in Table 2, after July 2014, Warfarin was consistently associated with significantly higher mortality compared to Aspirin. The hazard ratios increased to 2.181 (95% CI: 1.676 – 2.839, p < 0.001), indicating a statistically significant increase in mortality associated with Warfarin. For the analysis after July 2014, significant risk factors included Age (Over 65) (HR = 1.872, p < 0.001), Smoking (Heavy) (HR = 2.036, p < 0.001), Alcohol (Yes) (HR = 0.473, p < 0.001), Contraindications (Present) (HR = 0.521, p = 0.0144), Low molecular weight heparin (On medication) (HR = 1.585, p = 0.0029), NSAID (Present) (HR = 0.671, p = 0.0278), and ACE inhibitor use (Present) (HR = 0.471, p = 0.0079).

For the combined cohort including both pre- and post-July 2014 data, the hazard ratio was 1.863 (95% CI: 1.497 – 2.318, p < 0.001). These values closely mirror those observed in the post-July 2014 subgroup, suggesting that the overall cohort was heavily influenced by data collected after the trial’s completion. This highlights the potential for post-trial changes in treatment practices to dominate aggregated analyses. It further underscores the need to consider temporal stratification when interpreting real-world evidence.

These findings suggest that while no significant treatment difference was observed prior to WARCEF trial completion, a clear and statistically significant survival benefit favouring Aspirin emerged afterward. This temporal shift underscores the influence of clinical trial completion and dissemination on real-world treatment effectiveness. It also highlights how treatment effects may evolve over time in response to changing clinical practices, awareness, and decision-making processes. The divergence between pre- and post-trial periods suggests that real-world evidence is not static and underscores the importance of time-sensitive analyses in trial emulation studies. These results reinforce the need to consider temporal context when evaluating treatment outcomes and interpreting the broader impact of clinical trials beyond their publication.

**Fig. 1:**
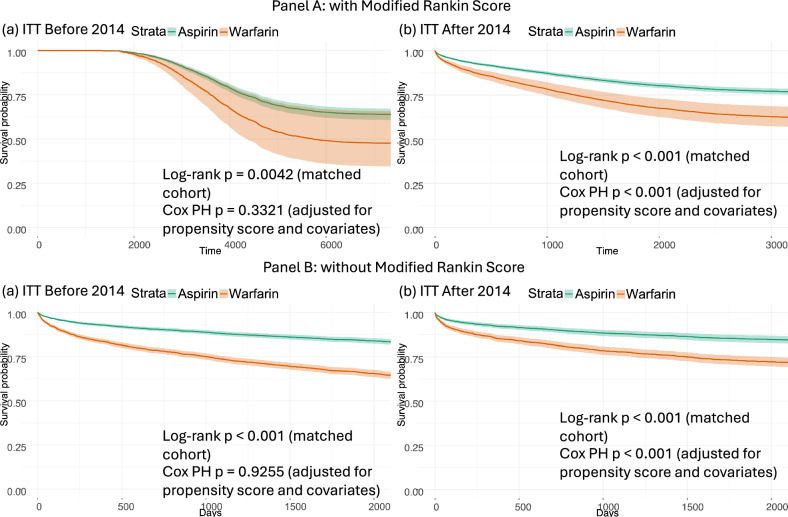
Survival curves stratified by analysis type and time period for ITT analysis with modified rankin score and without modified rankin score. This figure presents Kaplan–Meier survival curves comparing Aspirin (green) and Warfarin (orange) under ITT analyses. The plots are organized as follows: the **A** is the cohort with Modified Rankin Score while **B** is the cohort without Modified Rankin Score. The left column shows data from before 2014, and the right column shows data from after 2014. Prior to 2014, no significant difference in survival was observed between the two treatments. However, after 2014, a clear separation emerges between the curves, indicating a statistically significant survival benefit for patients treated with Aspirin. Across all stratifications, Aspirin consistently demonstrates lower mortality risk compared to Warfarin, particularly in the post-2014 period. To illustrate this difference (the flat area), we plotted all observed deaths in the cohort that included the Modified Rankin Score criterion, while applying a fixed 2190-day (6-year) follow-up window for the cohort without the Modified Rankin Score. The log-rank *p* value shown in each panel corresponds to the unadjusted comparison of survival distributions between treatment groups in the matched cohort. The Cox proportional hazards (PH) *p* value represents the treatment effect estimated from a multivariable Cox model adjusted for the propensity score and additional covariates. Differences between log-rank and Cox *p* values may arise because the Cox model accounts for covariate adjustment whereas the log-rank test reflects an unadjusted survival comparison.

As shown in Fig. 1 (a) of Panel A for the pre-2014 cohort, the survival curves display a pronounced flat region during the first ~1600 days of follow-up. This pattern arises from the use of the Modified Rankin Score as a strict inclusion criterion. Applying this criterion delays the effective cohort entry, such that all observed deaths occur after 2017. As a result, no events are observed in the early follow-up period, producing the initial plateau in the survival curves and influencing the estimated survival patterns and treatment effects.

As shown in Fig. 1 (a) of Panel B, removing the Modified Rankin Score from the inclusion criteria eliminates the early flat region, indicating that the previously observed plateau was driven by selection bias introduced by this criterion. The results indicate that Warfarin is associated with higher mortality compared to Aspirin. While the difference between the two treatments was not statistically significant before 2014, a significant divergence emerges after 2014, with Aspirin showing a clear survival advantage. The Survival curves for PP analysis is presented in Supplement Fig. 2 in the Supplementary Information.

**Fig. 2:**
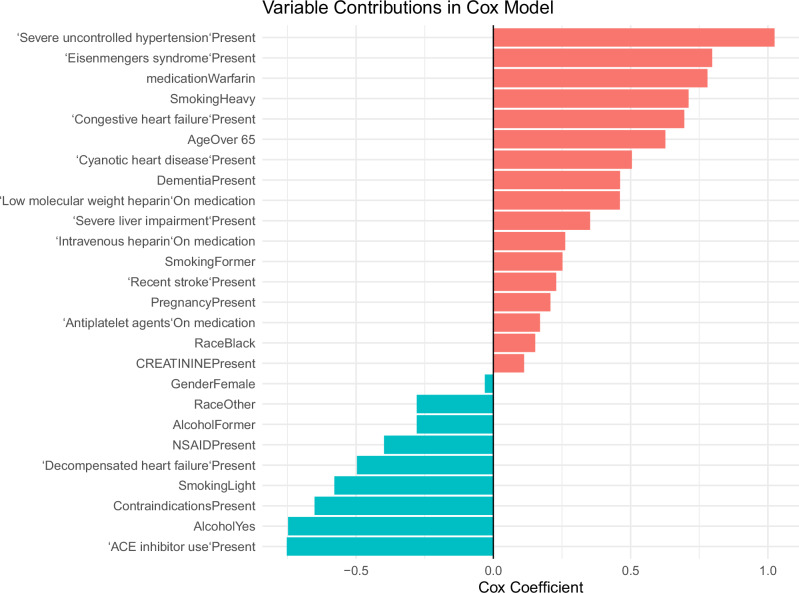
Confounders coefficient of ITT after 2014. This figure illustrates the most contributive clinical variables influencing outcomes in the ITT analysis conducted after 2014. The horizontal bar plot presents variable names along the y-axis and their corresponding coefficient values along the x-axis. Positive coefficients reflect stronger associations with increased outcome probability or risk, while negative coefficients indicate potential protective effects. Variables with positive coefficients indicate an association with increased hazard, while negative coefficients indicate a lower estimated hazard. Among the strongest positive contributors (higher risk) are severe uncontrolled hypertension, Eisenmenger’s syndrome, and Warfarin treatment, followed by heavy smoking, congestive heart failure, and older age (≥65 years), indicating that severe cardiovascular comorbidities and advanced age are major drivers of mortality risk in this cohort. Additional positive contributors include cyanotic heart disease, dementia, and anticoagulant-related medications (e.g., low-molecular-weight heparin and intravenous heparin), reflecting the influence of complex clinical severity and treatment context. Variables with smaller positive coefficients, such as recent stroke, pregnancy, antiplatelet use, race (Black), and elevated creatinine, contribute more modestly to risk. In contrast, several covariates exhibit negative coefficients (lower risk), including ACE inhibitor use, alcohol use (current and former), contraindications, light smoking, and decompensated heart failure, which may reflect treatment effects, residual confounding, or clinical selection factors rather than true protective effects. Other modest negative contributors include NSAID use and race categorized as “Other.” Overall, the figure demonstrates substantial heterogeneity in covariate effects, with severe cardiovascular conditions, treatment status, and age emerging as dominant determinants of mortality risk in the matched cohort.

Figure 2 displays the estimated Cox regression coefficients for clinical variables treated as confounders in the intention-to-treat analysis conducted after completion of the WARCEF trial. Positive coefficients indicate variables associated with higher hazard, whereas negative coefficients indicate inverse associations with the outcome. The largest positive contributors include severe uncontrolled hypertension, Eisenmenger’s syndrome, and warfarin therapy, followed by heavy smoking, congestive heart failure, and older age (≥65 years), highlighting the dominant role of severe cardiovascular comorbidities and advanced age in shaping risk in the post-trial population. Additional positive associations are observed for cyanotic heart disease, dementia, and anticoagulant-related medications such as low-molecular-weight and intravenous heparin, reflecting the influence of clinical severity and treatment indication. In contrast, several variables exhibit negative coefficients, including ACE inhibitor use, alcohol use (current and former), contraindications to therapy, light smoking, and decompensated heart failure, which may reflect clinical selection processes, treatment effects, or residual confounding rather than true protective effects. Overall, the figure demonstrates substantial heterogeneity in covariate associations following trial dissemination and underscores the importance of careful confounder control when emulating randomized trial effects using real-world post-trial data.

Supplement Figs. 3–5 in the Supplementary Information provide additional coefficient plots for the ITT analysis before 2014, as well as the PP analyses conducted before and after 2014.

Table 3 presents Cox proportional hazards results across multiple cutoff years using the downsampled dataset, employed to address substantial imbalance in patient counts before and after each cutoff when the Modified Rankin Score is incorporated, and the full dataset that excludes the Modified Rankin Score, with post-cutoff patient counts adjusted to match those before each cutoff. In analyses using the downsampled dataset, a clear temporal pattern emerges: cutoff years prior to 2013 show no significant differences between Warfarin and Aspirin, whereas cutoff years after 2015 yield statistically significant and progressively larger hazard ratios favoring Aspirin. The year 2014 represents a transition point, with estimates remaining non-significant before the cutoff and becoming significant thereafter, suggesting an apparent temporal shift in treatment effects. The consistency of this pattern across multiple cutoff years supports the use of 2014 as a practical boundary in analyses that include the Modified Rankin Score. In contrast, when the Modified Rankin Score is excluded, 2014 no longer appears to be a uniquely influential cutoff year. Across all evaluated cutoff years, estimates are generally non-significant before the cutoff and become significant afterward, irrespective of the specific year chosen. This pattern indicates that the observed differences are not attributable to 2014 itself but are instead driven by cohort definition. Accordingly, 2014 cannot be identified as the sole or definitive cutoff year in analyses that do not incorporate the Modified Rankin Score.

**Table 3:** Comparison of cox proportional hazards results across different cutoff dates with downsampled data and modified rankin score, and with full data and no modified rankin score

Cutoff Year	Analysis Period	Patient Population with Modified Rankin Score (Aspirin | Warfarin)	Hazard Ratio [HR], p-value	Patient Population without Modified Ranking Score (Aspirin | Warfarin)	Hazard Ratio [HR], p-value
2012	Before	2954 | 60	1.328 (p = 0.5212)	29,216 | 1661	0.813 (p = 0.5946)
	After	3052 | 96	1.641 (p = 0.2874)	34,128 | 1436	2.083 (p < 0.001)
2013	Before	3734 | 72	0.905 (p = 0.6891)	33,343 | 1847	1.004 (p = 0.9894)
	After	2889 | 93	1.693 (p = 0.156)	30,001 | 1250	2.266 (p < 0.001)
2014	Before	4576 | 93	1.3858 (p = 0.3321)	37,180 | 2019	1.025 (p = 0.9255)
	After	3343 | 122	1.972 (p = 0.0419)	26,164 | 1078	2.181 (p < 0.001)
2015	Before	5458 | 112	0.9704 (p = 0.9245)	40,586 | 2165	1.165 (p = 0.5842)
	After	3150 | 116	2.960 (p = 0.0027)	22,758 | 932	2.221 (p < 0.001)
2016	Before	6474 | 139	1.163 (p = 0.5663)	43,907 | 2309	1.064 (p = 0.8091)
	After	2868 | 114	2.311 (p = 0.0056)	19,437 | 788	2.156 (p < 0.001)
2017	Before	7710 | 170	1.215 (p = 0.998)	47,285 | 2436	1.133 (p = 0.5973)
	After	2621 | 112	3.127 (p = 0.0013)	16,059 | 661	2.200 (p < 0.001)

## Discussion

While our initial analyses identified differences in estimated treatment effects before and after 2014, subsequent investigations indicate that these differences are not primarily driven by temporal changes associated with the publication and dissemination of the WARCEF trial. Instead, the observed divergence arises largely from the application of specific eligibility criteria that substantially alter cohort composition and event timing. In particular, restrictive inclusion criteria, especially those based on functional status assessments, can delay effective risk entry and selectively exclude early events, thereby inducing selection bias that materially influences hazard ratio estimates. Importantly, the inclusion criteria did not require patients to survive beyond any fixed period, nor was a minimum follow-up time imposed at cohort entry. Nevertheless, several eligibility criteria relied on clinical assessments whose availability is inherently time-dependent in real-world practice. Although such criteria do not explicitly restrict patients by time or survival, their operationalization can implicitly induce delayed cohort entry and selection-induced truncation of early events. In our study, the requirement for a documented Modified Rankin Score exemplified this mechanism, producing an artificial early plateau in survival curves that resolved once the criterion was removed. This eligibility-driven selection bias can create the appearance of temporal heterogeneity even when underlying treatment effects remain stable. As a result, stratifying analyses solely by calendar time or trial completion date may obscure the true source of variation in emulated outcomes. Our findings suggest that eligibility definitions, rather than trial timing itself, play a dominant role in shaping observed treatment effects in real-world trial emulation. Taken together, these results underscore that accurate trial emulation requires not only temporal alignment with the original trial but, more critically, a principled evaluation of how eligibility criteria translate into real-world data. Overly restrictive or imperfectly operationalized criteria, even a single one, can disproportionately affect risk sets, follow-up duration, and inferred comparative effectiveness, potentially leading to misleading interpretations of post-trial treatment effects.

Although the timing of the observed post-2014 divergence coincides with the publication and dissemination of WARCEF, our analysis cannot determine whether this reflects causal effects of trial findings, shifts in treatment selection, or evolving clinical practice patterns. The results should therefore be interpreted as descriptive, hypothesis-generating evidence of temporal heterogeneity in comparative outcomes rather than as evidence of dissemination-driven risk differences.

Although the WARCEF trial reported a neutral primary outcome^11^, its publication likely amplified its visibility and influence on clinical decision-making. High-profile trials, even those with negative or neutral results, can shift practice when addressing key therapeutic uncertainties. Supporting this, Homma et al. ^16^. showed that among patients under 60 years old, Warfarin was associated with improved outcomes compared to Aspirin, while older patients saw no such benefit, indicating the interpretation clinicians may apply following publication. Furthermore, quality of anticoagulation control, measured by time in therapeutic range (TTR), was found to be a critical modifier of treatment efficacy: higher TTRs were significantly associated with better outcomes in Warfarin-treated patients^17^. These findings underscore how post-trial analyses can reinforce or refine clinical interpretations, further affecting treatment patterns.

Nonetheless, broader shifts in heart failure management, such as evolving clinical guidelines, new therapeutic options, and changes in care delivery, may also contribute to the observed treatment effect differences over time. These systemic developments can confound real-world analyses and must be considered alongside the influence of individual trial findings. Real-world emulations must therefore be contextualized within the broader landscape of clinical practice, which is continually evolving. Our findings are preliminary but underscore the importance of further research to understand how and when trial publications impact clinical behaviour, particularly across diverse health systems and patient populations. In addition, accurate trial emulation demands rigorous adherence to the original trial’s eligibility criteria, intervention definitions, and study timeline. Ensuring that the emulated patient cohort aligns precisely with the trial period is critical for producing valid and interpretable results.

Through the trial emulation process, we use Cox model^18^ to do the survival analysis. We found that the Cox model’s outputs are highly sensitive to how covariates are specified, particularly the selection and treatment of confounding variables. For instance, defining ACE inhibitor use as a strict inclusion criterion resulted in a HR below 1, suggesting a protective effect, whereas treating it as a confounder produced an HR above 1, implying potential harm. This discrepancy underscores a crucial methodological challenge: the outcome of emulated trials can vary substantially based on how variables are handled. To mitigate such inconsistencies, there is a pressing need for a principled framework, perhaps incorporating probabilistic modelling or data-driven selection criteria, to guide variable inclusion and classification. Such a framework would not only improve the reproducibility and interpretability of emulation studies but also reduce subjective bias. In our study, we worked closely with clinical experts to define confounders and ensure alignment with domain knowledge, reflecting a pragmatic approach in the absence of universally accepted standards.

A key limitation of this study lies in the observational nature of treatment assignment. Unlike the original WARCEF randomized controlled trial, medication exposure in our real-world emulation was determined by physicians’ prescribing practices rather than random allocation. As a result, unmeasured or unrecorded confounders, such as clinical judgment, patient adherence, socioeconomic status, comorbid burden, and subtle health status, may have influenced both treatment choice and outcomes. Although we applied propensity score–based adjustment to balance measured covariates and mitigate observable confounding, residual bias from unmeasured factors cannot be fully excluded. Moreover, clinical practice patterns, prescribing preferences, and access to care may have evolved over time, potentially introducing temporal confounding that further complicates interpretation. These limitations underscore the inherent challenge of approximating randomization using real-world data, where treatment decisions reflect individualized clinical reasoning rather than experimental control. Therefore, our findings should be interpreted as reflecting associations under real-world clinical practice rather than causal effects equivalent to those observed in a randomized trial. The Cox proportional hazards models uses a default complete-case approach, and observations with missing covariate values were excluded from the adjusted analysis, which may introduce potential selection bias if the missingness is not completely random. More advanced imputation strategies, such as multiple imputation or model-based imputation techniques, can be used in the future to mitigate this limitation.

Furthermore, this study is the lack of detailed information on anticoagulation management, particularly regarding the active monitoring of the international normalized ratio (INR) among patients treated with Warfarin. In the original WARCEF trial, Warfarin dosing was rigorously adjusted through scheduled INR testing to maintain a therapeutic range of 2.5–3.0, which balanced the prevention of thromboembolic events against the risk of major bleeding. This level of close monitoring was central to the trial’s internal validity, ensuring consistent exposure to the intended therapeutic effect. In contrast, our real-world emulation lacked access to sufficiently granular INR data; laboratory values were often missing, inconsistently recorded, or obtained at irregular intervals, preventing accurate reconstruction of dose adjustments or therapeutic control. As a result, patients categorized as receiving Warfarin in our observational data may have experienced variable and suboptimal anticoagulation levels, including periods of under- or over-anticoagulation that would not have occurred under trial supervision. This limitation likely contributed to the poorer outcomes observed for Warfarin in the post-trial period and underscores the inherent challenges of replicating tightly controlled clinical protocols using real-world electronic health record data. It also highlights a broader issue in trial emulation studies, namely, that incomplete representation of medication management dynamics can bias treatment effect estimates and obscure distinctions between protocol-driven efficacy and real-world effectiveness.

A key limitation underlying this interpretation is that our real-world data do not capture the clinical management dimension of warfarin therapy, including INR target intensity, monitoring frequency, and time-in-therapeutic-range. These factors are integral components of real-world “warfarin treatment,” and their absence fundamentally constrains how treatment exposure can be interpreted in our emulation. As a result, the observed post-2014 divergence should not be understood as a change in the intrinsic pharmacologic effect of warfarin. Instead, it plausibly reflects shifts in patient selection, anticoagulation management quality, or monitoring intensity in the post-trial era, elements that are not observable in our dataset. We did not include INR as a covariate because it is a highly dynamic, time-varying parameter with substantial intra-patient variability and irregular measurement intervals. In routine practice, patients often have numerous INR measurements over time, with dosing guided by dedicated anticoagulation clinics and frequent adjustments to maintain therapeutic range. Consequently, a single baseline INR value or summary statistic would inadequately represent anticoagulation quality and could introduce misclassification or bias. In the absence of standardized longitudinal INR data required to consistently model time-in-therapeutic-range across patients, incorporating INR as a static covariate was not feasible in this analysis.

Another important limitation concerns the differences in background medication use between the pre-2014 and post-2014 cohorts. Over the past decade, the standard of care for patients with heart failure and reduced ejection fraction (HFrEF) has evolved substantially, with the increasing adoption of evidence-based therapies such as angiotensin receptor–neprilysin inhibitors (ARNIs) and sodium–glucose cotransporter 2 inhibitors (SGLT2i), alongside greater optimization of conventional agents including angiotensin-converting enzyme inhibitors (ACEIs) and angiotensin receptor blockers (ARBs). These advancements in pharmacotherapy may have significantly influenced overall patient outcomes and could confound the observed differences in treatment effects between time periods. Our method also has several other limitations. First, our analysis is restricted to a single clinical trial, and specifically to a trial with a negative primary outcome. This narrow scope may introduce selection bias and limit the generalizability of our findings. Second, the analysis was conducted exclusively on Mayo Clinic data, which may differ in demographic or clinical characteristics from other healthcare systems, potentially affecting the external validity of our results. Third, we used the trial’s official completion date as the temporal cutoff for cohort separation. However, this may not accurately capture the true timing of the trial’s influence on clinical practice, as the dissemination of results and subsequent adoption by different institutions may occur after publication and vary across sites.

## Methods

### Overview of the Proposed Method

**Fig. 3:**
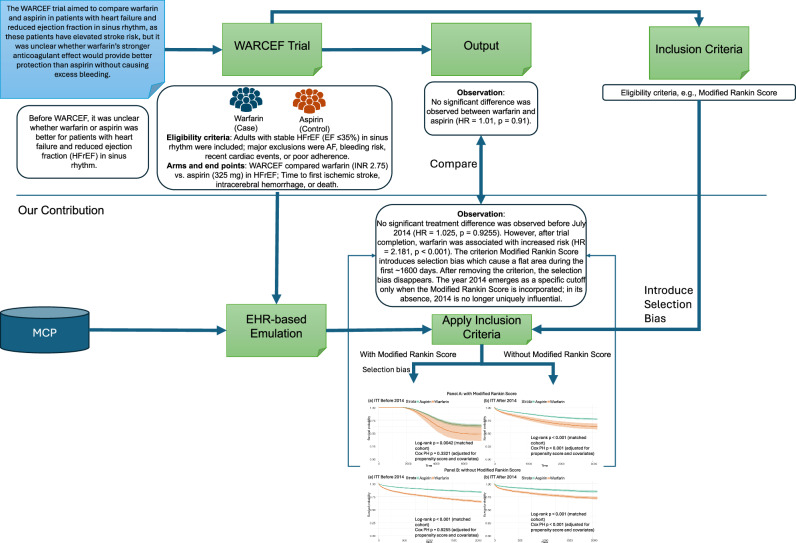
Overview of the proposed method. This figure summarizes the WARCEF trial and its EHR-based emulation. The WARCEF trial evaluated Warfarin versus Aspirin in patients with HFrEF in sinus rhythm, finding no significant difference in overall outcomes: Warfarin reduced ischemic stroke but increased bleeding, leading to guideline recommendations favouring Aspirin. To emulate the trial, real-world EHR data were used to construct a comparable patient cohort based on WARCEF’s inclusion and exclusion criteria. The emulation revealed a key finding. When the Modified Rankin Score was applied, cohort composition and event timing changed substantially: prior to 2014, the earliest observed death occurred in 2017, and no statistically significant difference between Warfarin and Aspirin was observed before the trial’s completion in July 2014, consistent with the original WARCEF findings. In contrast, a significant difference emerged after July 2014, initially suggesting that dissemination of the WARCEF results may have influenced clinical practice and outcomes in the broader patient population. However, when the Modified Rankin Score was excluded, 2014 no longer emerged as a uniquely influential cutoff year, indicating that the apparent temporal pattern was driven by eligibility criteria rather than the cutoff date itself.

Figure 3 outlines the overall workflow of our emulation study. It begins with the emulation of the WARCEF trial using real-world EHR data, where patient cohorts are constructed based on eligibility criteria. The figure highlights stratification by inclusion criterion, to examine potential shifts in treatment effect. In our emulation, patient cohorts were constructed using the Mayo Clinic Platform based on key eligibility criteria and refined to ensure comparability between treatment groups. Patients were categorized into Warfarin and Aspirin groups. All outcomes were extracted from electronic health records using definitions aligned with prior clinical trials. In our analysis, we used death as the outcome because the WARCEF trial also conducted a secondary analysis focused on mortality only. To enhance feasibility and ensure analytic consistency with the WARCEF trial, we adopted an intention-to-treat (ITT) approach^19,20^. The ITT approach includes all participants in their originally assigned treatment groups, regardless of adherence, thereby preserving the benefits of randomization and minimizing selection bias. This method offers a realistic estimate of treatment effectiveness in typical real-world conditions. Patients were further stratified by with or without Modified Rankin Score, to assess the potential impact of the inclusion criterion.

### Comparison of RCT and Target Trial Designs

Table 4 provides a detailed comparison between the traditional RCT design and our target trial emulation approach. It outlines key aspects, including study aim, eligibility criteria, treatment strategies, follow-up period, and outcome assessment. This comparison highlights the methodological differences, particularly in how the target trial emulation leverages RWD to approximate an RCT while addressing feasibility and generalizability in clinical research. To align with the original WARCEF protocol, we systematically mapped the trial’s inclusion and exclusion criteria to structured EHR variables available within the Mayo Clinic Platform. Each criterion was classified as a must-include, must-exclude, or confounder based on its interpretability, availability, and influence on treatment assignment and outcome. Among the inclusion criteria, left ventricular ejection fraction (LVEF ≤ 35%) or wall motion index (WMI ≤ 1.2) measured within three months in a stable clinical state was treated as a strict inclusion criterion and applied without exception. In contrast, other criteria such as LVEF timing beyond 3 months post-intervention (e.g., CABG, PTCA), outpatient protocol adherence, and discharge status after IV CHF were not considered due to lack of structured availability or feasibility in routine data. The Modified Rankin score ( < 4) was used as a confounder, acknowledging its potential association with both treatment decisions and outcomes. Similarly, recent stroke or transient ischemic attack (TIA) within 12 months and ACE inhibitor use (or substitute therapies) were treated as confounders to preserve patient volume and statistical power while adjusting for their prognostic relevance.

**Table 4:** Comparison between Randomized Clinical Trial and Our Target Trial Design

Trials	Randomized clinical trial (NCT00041938)	Our target trial design
Aim	Warfarin Versus Aspirin in Reduced Cardiac Ejection Fraction (WARCEF) Trial (WARCEF)	Determine whether Warfarin or Aspirin is better for preventing death or stroke in patients with poor heart function
Eligibility criteria	**Inclusion criteria:**	**Criteria strictly included:**
	Cardiac EF < = 35% by radionuclide ventriculography, left ventriculography or quantitive echocardiographic measurement or an echocardiographic Wall Motion Index of <=1.2, within three months of enrollment. The qualifying left ventricular function measurement must be obtained at least three months after an MI, coronary bypass grafting, PTCA, and at least one month after pacemaker insertion. Patients scheduled for mitral valve repair should have qualifying echo after surgery.	Patients with Cardiac EF < = 35% (<=35% is used as a covariate in analysis) by radionuclide ventriculography, left ventriculography or quantitive echocardiographic measurement or an echocardiographic Wall Motion Index of <=1.2( < = 1.2 is used as a covariate in analysis), within three months of enrollment. The qualifying left ventricular function measurement must be obtained at least three months after an MI, coronary bypass grafting, PTCA, and at least one month after pacemaker insertion.
		**Criteria used as confounders:**
	Modified Rankin score <=4.	Modified Rankin score <=4 or Rankin score <=4
	Patient must be taking ACE inhibitors. If intolerant of ACE inhibitor, patient must be on angiotensin II receptor blockers or hydralazine and nitrates.	Patients must be taking ACE inhibitors. If intolerant of ACE inhibitor, patient must be on angiotensin II receptor blockers or hydralazine and nitrates.
	Recent stroke/TIA within 12 months	Recent stroke/TIA within 12 months
	**Exclusion criteria:**	**Criteria strictly excluded:**
	The presence of any of the following embolism: chronic or paroxysmal AF, mechanical valve, endocarditis, intracardiac mobile or pedunculated thrombus, and valvular vegetation.	The presence of any of the following embolism: chronic or paroxysmal AF, mechanical valve, endocarditis, intracardiac mobile or pedunculated thrombus, and valvular vegetation.
	Cardiac surgery/angioplasty/MI within 3 months.	Cardiac surgery/angioplasty/MI within 3 months.
	Cerebral/systemic hemorrhage in past year	Cerebral/systemic hemorrhage in past year.
	Age <18	Age <18
		**Criteria used as confounders:**
	Cyanotic congenital heart disease, Eisenmenger’s syndrome.	Cyanotic congenital heart disease, Eisenmenger’s syndrome.
	Decompensated heart failure.	Decompensated heart failure.
	Contraindications (e.g., GI bleed, INR > 1.3, low platelets, Hct<30).	Contraindications (e.g., GI bleed, INR > 1.3, low platelets, Hct<30).
	NSAID use.	NSAID use.
	Alcohol/substance abuse, gait instability.	Alcohol/substance abuse, gait instability.
	Severe liver impairment.	Severe liver impairment.
	Severe uncontrolled hypertension.	Severe uncontrolled hypertension.
	Positive stool guaiac (non-hemorrhoidal).	Positive stool guaiac (non-hemorrhoidal).
	Creatinine >3.0.	Creatinine >3.0.
	Need for heparin/antiplatelet agent.	Need for heparin/antiplatelet agent
	Dementia or psychiatric or physical problem that prevents the patient from following an outpatient program reliably.	Dementia or psychiatric or physical problem that prevents the patient from following an outpatient program reliably.
	Comorbid conditions that may limit survival to less than five years.	Comorbid conditions that may limit survival to less than five years.
	Pregnancy, or female of childbearing potential who is not sterilized or is not using a medically accepted form of contraception* (see procedure manual). *A pregnancy test is required for all women of childbearing age.	Pregnancy, or female of childbearing potential who is not sterilized or is not using a medically accepted form of contraception* (see procedure manual). *A pregnancy test is required for all women of childbearing age.
	Hospitalization for new diagnosis of onset CHF within the past one month or carotid endarterectomy or pacemaker insertion within the past one month prior to randomization.	Hospitalization for new diagnosis of onset CHF within the past one month or carotid endarterectomy or pacemaker insertion within the past one month prior to randomization.
Treatment strategies	Treatment 1: Aspirin (325 mg per day). Treatment 2: Warfarin [International Normalized Ration (INR) 2.5-3.0; target INR = 2.75].	Cohorts selected with medication administered: Aspirin (Dose is used as a covariate in analysis). Warfarin (INR is used as a covariate in analysis).
Treatment assignment	Randomized	Propensity score matching and inverse probability of treatment weighting.
Follow-up period	2002.10 -- 2011.08	Time Zero: First occurrence of all eligibility criteria satisfied. Follow-up: Up to 6 years from Time Zero
Outcome	Composite Endpoint of Ischemic Stroke, Intracerebral Hemorrhage, or Death.	Endpoint of Death.

Regarding exclusion criteria, several conditions were handled as strict exclusion filters, including: (1) Chronic/paroxysmal atrial fibrillation, mechanical valve, endocarditis, thrombus, or vegetation; (2) Cardiac surgery, angioplasty, or myocardial infarction within 3 months; (3) Cerebral or systemic hemorrhage within the past year; (4) Age < 18 years. These were directly excluded to ensure fidelity to trial conditions and minimize major sources of heterogeneity. Other clinical conditions that may influence both treatment and prognosis were modelled as confounders rather than hard exclusions. These included: (1) Cyanotic congenital heart disease and Eisenmenger’s syndrome; (2) Decompensated heart failure; (3) Contraindications to treatment (e.g., GI bleeding, abnormal INR or platelet counts, low hematocrit); (4) Use of NSAIDs; (5) Severe liver impairment, uncontrolled hypertension, elevated creatinine levels, and positive stool guaiac tests; (6) Pregnancy, dementia, and psychiatric/physical disability; (7) History of substance abuse, short life expectancy, lack of contraception, or recent CHF-related procedures. While the protocol called for strict exclusion of these patients, they were instead retained and statistically adjusted for to avoid excessive sample loss and maintain analytic robustness. Finally, certain exclusion criteria, such as “need for heparin/antiplatelet agent”, were retained in the cohort but treated as confounders due to the extremely small number of affected patients, making exclusion infeasible. A small number of criteria, such as “life expectancy <5 years” or contraception status, were not considered due to their absence or ambiguity in structured EHR data.

## Supplementary information

Supplementary information

## Data Availability

This study involves analysis of de-identified Electronic Health Record (EHR) data via Mayo Clinic Platform Discover. In accordance with the Code of Federal Regulations, 45 CFR 46.102, the noted activity does not require IRB review. Data shown and reported in this manuscript has been extracted from the EHR using an established protocol for data extraction, aimed at preserving patient privacy. The data has been determined to be de-identified pursuant to an expert’s determination in accordance with the HIPAA Privacy Rule. Any data beyond what is reported in the manuscript, including but not limited to the raw EHR data, cannot be shared or released due to the parameters of the expert determination to maintain the data de-identification. Contact corresponding authors for additional details regarding Mayo Clinic Platform Discover.

## Code availability

This study involves analysis of de-identified Electronic Health Record (EHR) data via Mayo Clinic Platform Discover. In accordance with the Code of Federal Regulations, 45 CFR 46.102, the noted activity does not require IRB review. Data shown and reported in this manuscript has been extracted from the EHR using an established protocol for data extraction, aimed at preserving patient privacy. The data has been determined to be de-identified pursuant to an expert’s determination in accordance with the HIPAA Privacy Rule. Any data beyond what is reported in the manuscript, including but not limited to the raw EHR data, cannot be shared or released due to the parameters of the expert determination to maintain the data de-identification. Contact corresponding authors for additional details regarding Mayo Clinic Platform Discover.
