# Supplementary material for: From “negative” trial to positive clinical impact: mitigating eligibility criteria–induced temporal selection bias in emulated clinical trials
Source: Npj Health Syst. 2026 May 27;3:36. doi: 10.1038/s44401-026-00082-3 (PMC13216059; doi:10.1038/s44401-026-00082-3)
Supplement: Supplementary file 1 — Supplementary information [file 44401_2026_82_MOESM1_ESM.pdf]

## Supplementary Information

In addition to the Cox proportional hazards analysis, we examined the temporal stability of the estimated effects over time. Supplement Table 1 summarizes the results of the Schoenfeld residuals test for the proportional hazards assumption applied to the medication variable in the Cox model, separately for the pre-2014 and post-2014 intention-to-treat (ITT) cohorts. The chi-square statistics were 0.00751 (p = 0.93094) before 2014 and 2.8717 (p = 0.09015) after 2014, indicating no significant deviation from the proportional hazards assumption in either period. These results suggest that the relative effect of medication on mortality risk remained time-invariant within both cohorts, supporting the validity of using a standard Cox proportional hazards model for estimating treatment effects before and after the trial dissemination.

**Supplement Table 1.** Schoenfeld residuals test for the proportional hazards assumption of the medication variable in pre- and post-2014 ITT cohorts.

| | ITT Before 2014 | ITT After 2014 |
|---|---|---|
| Chi-square | 0.00751 | 2.8717 |
| p-value | 0.93094 | 0.09015 |

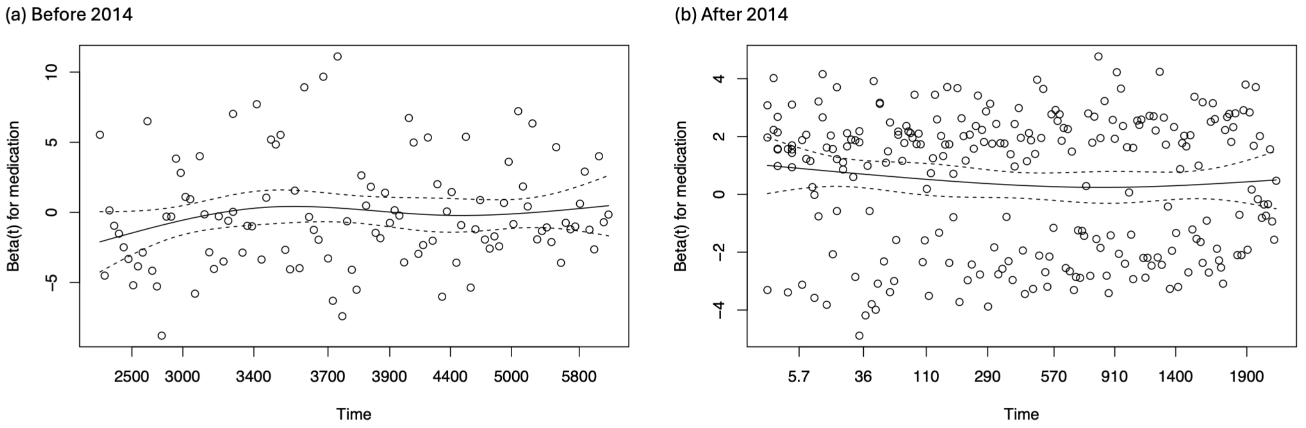


**Supplement Figure 1.** Schoenfeld residual plot for the medication variable from the Cox proportional hazards model Before and After 2014. The solid line represents the smoothed estimate of time-varying effects (β(t)), and the dashed lines denote the 95% confidence bands. The near-horizontal pattern of the smoothed curve indicates no systematic trend over time, suggesting that the proportional hazards assumption holds for the medication variable and that its effect on the hazard remains stable throughout the follow-up period.

**Supplement Table 2.** Comparison of Cox Proportional Hazards and RMST Model Results.

| Analysis Period | Cox Model (Hazard Ratio [HR], p-value) | RMST Difference (Arm 1 – Arm 0, p-value) |
|---|---|---|
| Before 2014 | 1.025 (p = 0.9255) | 4.8954 (p = 0.5236) |
| After 2014 | 2.181 (p < 0.001) | -217.5866 (p = 4.3170e-10) |

Supplement Table 2 summarizes the results from both the Cox proportional hazards and RMST models. The analyses reveal a consistent pattern: cutoff years prior to 2014 show no statistically significant differences between treatment groups, whereas results after 2014 become highly significant across both models. This alignment between the Cox and RMST findings reinforces the validity of the proportional hazards model and highlights 2014 as a clear inflection point in treatment effects.

**Supplement Table 3.** Cox Proportional Hazards Model Results for the Integrated Population Including Cohort Indicator (pre- vs post-2014).

| | Medication (Warfarin) | Cohort Indicator (After 2014) as a Confounder |
|---|---|---|
| HR | 1.365 (1.075 – 1.734) | 36.311 (21.106 – 62.471) |
| p-value | 0.0108 | <0.001 |

Supplement Table 3 presents the results of a Cox proportional hazards model incorporating both treatment (Warfarin vs Aspirin) and cohort (pre- vs post-2014) indicators to evaluate whether the temporal shift observed after July 2014 persists when both groups are analyzed jointly. The hazard ratio for Warfarin was 1.365 ($p = 0.0108$), indicating an increased risk of mortality relative to Aspirin when controlling for cohort effects. The cohort indicator (After 2014) also showed a significant association ($p < 0.001$), suggesting a notable change in outcome distributions following the trial dissemination. These findings confirm that the post-2014 increase in mortality risk for Warfarin-treated patients is not merely an artifact of sample size imbalance before 2014 but reflects a genuine divergence between pre- and post-trial clinical practice patterns.

**Supplement Table 4.** Hazard Ratios for Mortality Across Both Significant and Non-significant Risk Factors.

ITT Before July 2014

| Medication (Warfarin) | | |
|---|---|---|
| Hazard Ratio | | p-value |
| 1.025 (0.6144 – 1.709) | | 0.9255 |
| Risk Factors | | |
| RF | Hazard Ratio | p-value |
| Age (Over 65) | 2.894 (1.844 – 4.543) | <0.001 |
| Gender (Female) | 0.844 (0.530 – 1.345) | 0.476 |
| Race (Black) | 0.622 (0.218 – 1.775) | 0.3744 |
| Race (Other) | 0.969 (0.298 – 3.155) | 0.958 |
| Smoking (Heavy) | 2.613 (1.279 – 5.337) | 0.0084 |
| Smoking (Light) | 1.002 (0.231 – 4.351) | 0.9983 |
| Smoking (Former) | 1.208 (0.768 – 1.901) | 0.413 |
| Alcohol (Yes) | 0.470 (0.273 – 0.808) | 0.0063 |

ITT After July 2014

| Medication (Warfarin) | | |
|---|---|---|
| Hazard Ratio | | p-value |
| 2.181 (1.676 – 2.839) | | <0.001 |
| Risk Factors | | |
| RF | Hazard Ratio | p-value |
| Age (Over 65) | 1.872 (1.412 – 2.481) | <0.001 |
| Gender (Female) | 0.969 (0.737 – 1.276) | 0.8244 |
| Race (Black) | 1.165 (0.734 – 1.848) | 0.5167 |
| Race (Other) | 0.757 (0.380 – 1.504) | 0.4262 |
| Smoking (Heavy) | 2.036 (1.340 – 3.095) | <0.001 |
| Smoking (Light) | 0.560 (0.176 – 1.781) | 0.3262 |
| Smoking (Former) | 1.287 (0.966 – 1.713) | 0.0847 |
| Alcohol (Yes) | 0.473 (0.324 – 0.691) | <0.001 |

| | | |
|---|---|---|
| Alcohol (Former) | 0.509 (0.195 – 1.332) | 0.169 |
| Contraindications (Present) | 0.644 (0.370 – 1.123) | 0.1208 |
| Cyanotic heart disease (Present) | 0.720 (0.0897 – 5.782) | 0.7574 |
| Eisenmengers syndrome (Present) | 1.385 (0.465 – 4.126) | 0.5591 |
| Decompensated heart failure (Present) | 1.089 (0.425 – 2.792) | 0.8595 |
| Intravenous heparin (On medication) | 1.789 (0.504 – 6.345) | 0.3681 |
| Low molecular weight heparin (On medication) | 1.663 (1.052 – 2.630) | 0.0296 |
| Dementia (Present) | 1.223 (0.691 – 2.165) | 0.4899 |
| Pregnancy (Present) | 0.955 (0.474 – 1.926) | 0.8981 |
| Congestive heart failure (Present) | 1.556 (0.625 – 3.872) | 0.3423 |
| Carotid endarterectomy (Present) | 3.048 (0.380 – 24.428) | 0.294 |
| NSAID (Present) | 0.938 (0.606 – 1.452) | 0.7742 |
| Severe liver impairment (Present) | 5.052 (0.666 – 38.326) | 0.1172 |
| Severe uncontrolled hypertension (Present) | 2.315 (0.520 – 10.310) | 0.2707 |
| CREATININE (Present) | 0.766 (0.176 – 3.337) | 0.7229 |
| ACE inhibitor use (Present) | 0.865 (0.307 – 2.437) | 0.7835 |
| Recent Stroke (Present) | 0.871 (0.260 – 2.921) | 0.823 |

| | | |
|---|---|---|
| Alcohol (Former) | 0.756 (0.505 – 1.134) | 0.1769 |
| Contraindications (Present) | 0.521 (0.309 – 0.879) | 0.0144 |
| Cyanotic heart disease (Present) | 1.656 (0.220 – 12.477) | 0.6243 |
| Eisenmengers syndrome (Present) | 2.219 (0.793 – 6.209) | 0.1289 |
| Decompensated heart failure (Present) | 0.608 (0.246 – 1.502) | 0.2812 |
| Intravenous heparin (On medication) | 1.299 (0.912 – 1.852) | 0.1475 |
| Low molecular weight heparin (On medication) | 1.585 (1.171 – 2.146) | 0.0029 |
| Antiplatelet agents (On medication) | 1.185 (0.569 – 2.471) | 0.6499 |
| Dementia (Present) | 1.587 (0.949 – 2.653) | 0.0783 |
| Pregnancy (Present) | 1.231 (0.756 – 2.004) | 0.4031 |
| Congestive heart failure (Present) | 2.005 (0.905 – 4.441) | 0.0864 |
| NSAID (Present) | 0.671 (0.471 – 0.958) | 0.0278 |
| Severe liver impairment (Present) | 1.422 (0.943 – 2.146) | 0.0932 |
| Severe uncontrolled hypertension (Present) | 2.786 (0.986 – 7.874) | 0.0533 |
| CREATININE (Present) | 1.119 (0.715 – 1.749) | 0.6236 |
| ACE inhibitor use (Present) | 0.471 (0.270 – 0.821) | 0.0079 |
| Recent Stroke (Present) | 1.257 (0.445 – 3.555) | 0.6658 |

PP Before July 2014

| Medication (Warfarin) | |
|---|---|
| Hazard Ratio | p-value |

PP After July 2014

| Medication (Warfarin) | |
|---|---|
| Hazard Ratio | p-value |

| | |
|---|---|
| 0.398 (0.1098 – 1.441) | 0.1605 |

| Risk Factors | | |
|---|---|---|
| RF | Hazard Ratio | p-value |
| Age (Over 65) | 12.193 (3.613 – 41.150) | <0.001 |
| Gender (Female) | 1.623 (0.546 – 4.830) | 0.3839 |
| Smoking (Heavy) | 2.198 (0.351 – 13.752) | 0.3998 |
| Smoking (Light) | 10.872 (0.757 – 156.246) | 0.0793 |
| Smoking (Former) | 0.792 (0.261 – 2.401) | 0.6801 |
| Alcohol (Yes) | 0.681 (0.184 – 2.524) | 0.5658 |
| Contraindications (Present) | 0.325 (0.076 – 1.385) | 0.1286 |
| Decompensated heart failure (Present) | 1.558 (0.137 – 17.742) | 0.7207 |
| Low molecular weight heparin (On medication) | 2.813 (0.930 – 8.511) | 0.067 |
| Dementia (Present) | 5.276 (1.541 – 18.073) | 0.0081 |
| Pregnancy (Present) | 1.167 (0.208 – 6.558) | 0.8608 |
| Congestive heart failure (Present) | 0.112 (0.011 – 1.111) | 0.0615 |
| NSAID (Present) | 0.710 (0.216 – 2.333) | 0.5721 |
| ACE inhibitor use (Present) | 0.157 (0.027 – 0.925) | 0.0407 |
| Recent stroke (Present) | 1.453 (0.084 – 25.059) | 0.7972 |

| | |
|---|---|
| 3.087 (1.8171 – 5.243) | <0.001 |

| Risk Factors | | |
|---|---|---|
| RF | Hazard Ratio | p-value |
| Age (Over 65) | 0.923 (0.504 – 1.690) | 0.7955 |
| Gender (Female) | 1.112 (0.629 – 1.965) | 0.7157 |
| Race (Black) | 4.217 (1.926 – 9.232) | <0.001 |
| Race (Other) | 3.020 (1.034 – 8.819) | 0.0432 |
| Smoking (Heavy) | 0.660 (0.293 – 1.483) | 0.3142 |
| Smoking (Light) | 0.431 (0.056 – 3.290) | 0.4167 |
| Smoking (Former) | 1.259 (0.737 – 2.151) | 0.3995 |
| Alcohol (Yes) | 0.799 (0.394 – 1.622) | 0.5347 |
| Alcohol (Former) | 3.182 (1.436 – 7.051) | 0.0044 |
| Contraindications (Present) | 0.768 (0.318 – 1.853) | 0.557 |
| Eisenmengers syndrome (Present) | 0.550 (0.150 – 2.019) | 0.3672 |
| Decompensated heart failure (Present) | 1.203 (0.219 – 6.595) | 0.8315 |
| Intravenous heparin (On medication) | 2.005 (0.845 – 4.761) | 0.1147 |
| Low molecular weight heparin (On medication) | 1.067 (0.629 – 1.807) | 0.8106 |
| Antiplatelet agents (On medication) | 1.470 (0.653 – 3.307) | 0.3522 |
| Dementia (Present) | 0.820 (0.275 – 2.446) | 0.7212 |
| Pregnancy (Present) | 0.634 (0.254 – 1.579) | 0.3272 |
| Congestive heart failure (Present) | 2.460 (0.595 – 10.168) | 0.2136 |
| NSAID (Present) | 0.892 (0.466 – 1.706) | 0.7294 |
| Severe liver impairment (Present) | 0.931 (0.368 – 2.352) | 0.879 |
| Severe uncontrolled hypertension (Present) | 1.279 (0.288 – 5.684) | 0.7465 |
| CREATININE (Present) | 0.600 (0.182 – 1.979) | 0.4018 |
| ACE inhibitor use (Present) | 0.367 (0.169 – 0.797) | 0.0113 |
| Recent Stroke (Present) | 1.111 (0.202 – 6.122) | 0.9036 |

Supplement Table 4 summarizes the Cox model–estimated hazard ratios and corresponding p-values for all covariates evaluated in the mortality analysis, including both significant and non-significant factors. This table was added to provide full transparency regarding the covariate assessment described in Section 2.2, clarifying that these analyses served a supportive role to contextualize treatment effects rather than to identify new predictors of mortality.

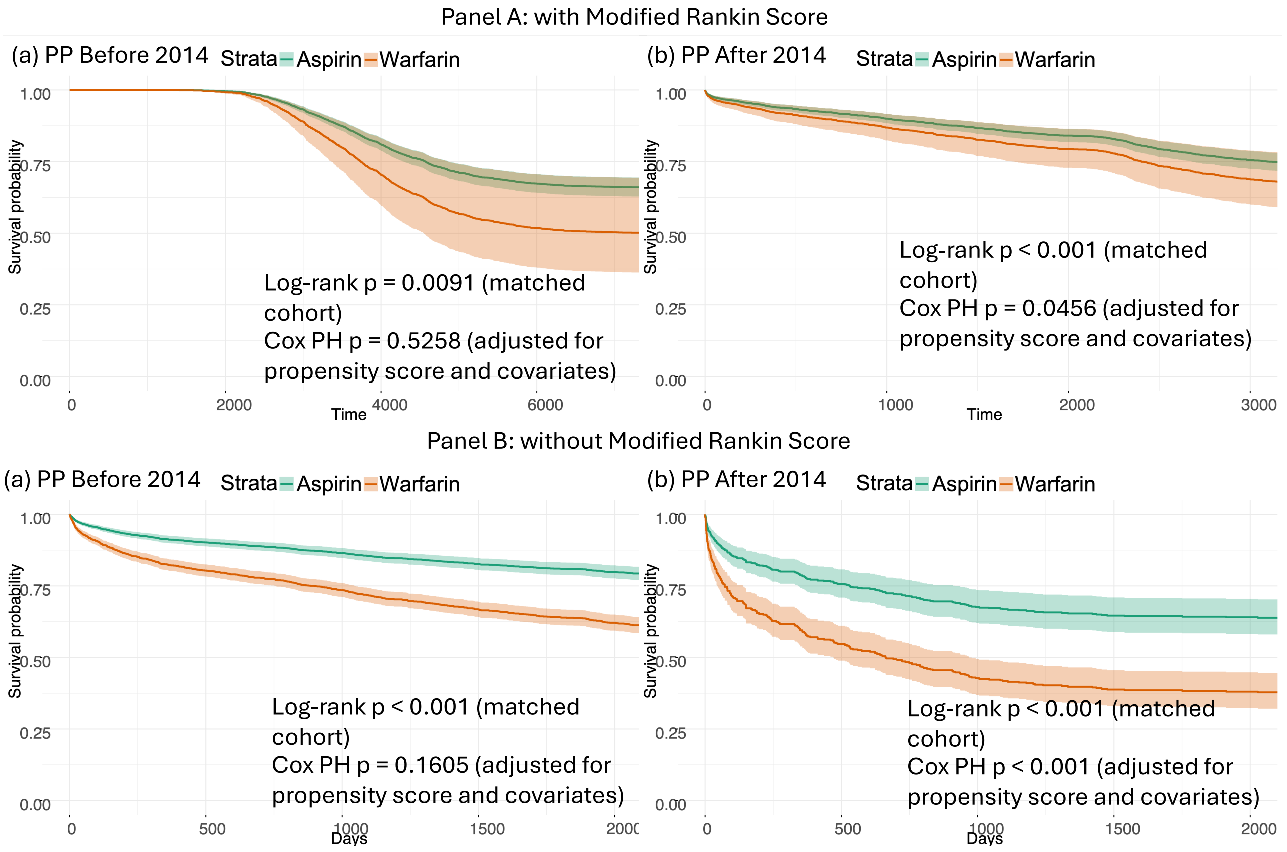


**Supplement Figure 2.** Survival Curves Stratified by Analysis Type and Time Period for PP Analysis with Modified Rankin Score and without Modified Rankin Score. This figure presents Kaplan–Meier survival curves comparing Aspirin (green) and Warfarin (orange) under PP analyses. The plots are organized as follows: the Panel A is the cohort with Modified Rankin Score while Panel B is the cohort without Modified Rankin Score. The top row shows data from before 2014, and the bottom row shows data from after 2014. Prior to 2014, no significant difference in survival was observed between the two treatments. However, after 2014, a clear separation emerges between the curves, indicating a statistically significant survival benefit for patients treated with Aspirin. Across all stratifications, Aspirin consistently demonstrates lower mortality risk compared to Warfarin, particularly in the post-2014 period. The log-rank p-value shown in each panel corresponds to the unadjusted comparison of survival distributions between treatment groups in the matched cohort. The Cox proportional hazards (PH) p-value represents the treatment effect estimated from a multivariable Cox model adjusted for the propensity score and additional covariates. Differences between log-rank and Cox p-values may arise because the Cox model accounts for covariate adjustment whereas the log-rank test reflects an unadjusted survival comparison.

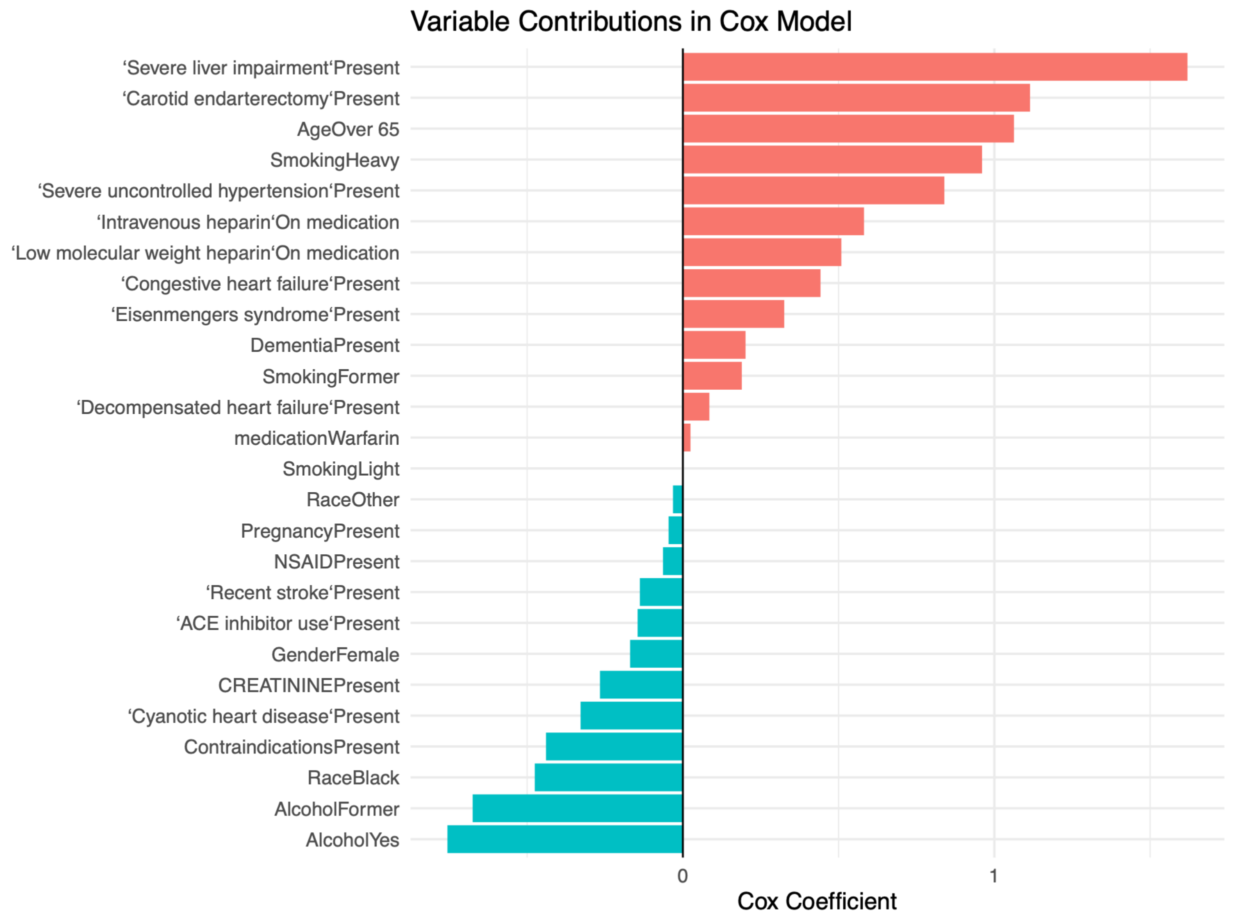


**Supplement Figure 3.** Confounders Coefficient of ITT before 2014. This figure displays the most influential clinical variables contributing to the outcome in the ITT analysis conducted prior to 2014. The horizontal bar plot ranks variables by the magnitude of their regression coefficients, with variable names listed on the y-axis and their corresponding coefficients on the x-axis. Positive coefficients indicate a stronger association with increased risk or likelihood of the outcome, whereas negative coefficients reflect a protective effect or decreased risk. Several cardiovascular and procedure-related variables show the strongest positive associations (higher mortality risk) in the pre-2014 cohort, most notably severe liver impairment and carotid endarterectomy, followed by older age (≥65 years), heavy smoking, severe uncontrolled hypertension, and anticoagulant use (intravenous and low-molecular-weight heparin). Additional positive contributors include congestive heart failure, Eisenmenger's syndrome, dementia, and former smoking, indicating that advanced systemic disease burden and prior cardiovascular intervention were major drivers of risk before trial completion. Warfarin therapy shows only a modest positive coefficient relative to these clinical factors. In contrast, several variables exhibit negative coefficients (lower associated risk), including alcohol use (current and former), race categorized as Black, contraindications to therapy, cyanotic heart disease, elevated creatinine, female sex, ACE inhibitor use, and recent stroke. These inverse associations likely reflect clinical selection, survivorship effects, or residual confounding inherent to observational data rather than true protective effects. Overall, the figure demonstrates marked heterogeneity in covariate associations in the pre-2014 population and highlights the dominant influence of severe comorbidity and prior intervention patterns on mortality risk before dissemination of trial findings.

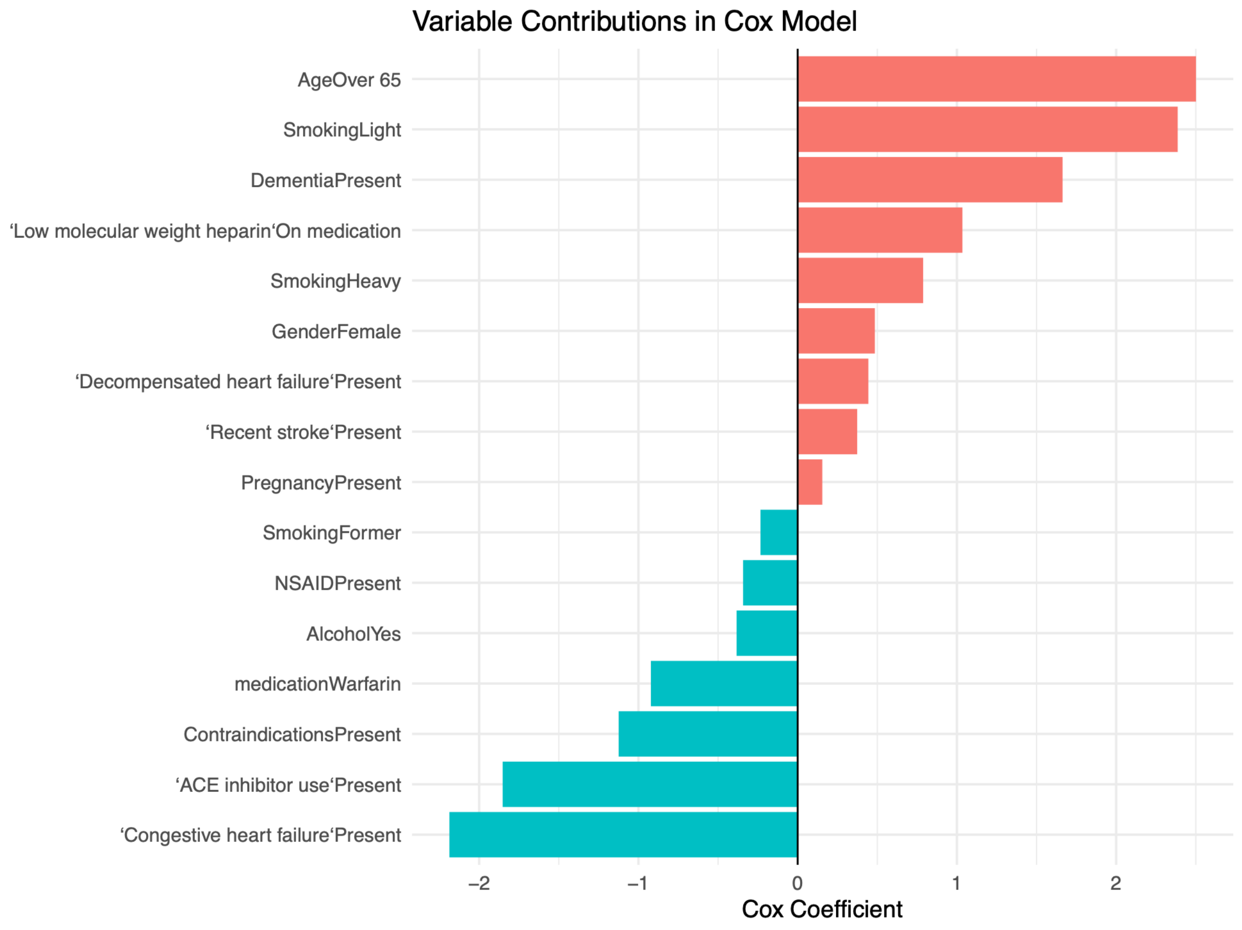


**Supplement Figure 4.** Confounders Coefficient of PP before 2014. This figure presents the most contributive clinical variables associated with outcomes in the PP analysis prior to 2014. The horizontal bar chart displays clinical variables on the y-axis and their corresponding coefficient values on the x-axis. Positive coefficients indicate an increased risk or likelihood of the outcome, while negative coefficients suggest a protective effect or reduced risk. The largest positive associations with mortality risk in the pre-2014 per-protocol cohort are observed for older age (≥65 years), light smoking, and dementia, followed by low-molecular-weight heparin use, heavy smoking, female sex, decompensated heart failure, and recent stroke. These findings indicate that advanced age, neurologic comorbidity, smoking behavior, and markers of clinical severity were major drivers of risk in this population. Pregnancy shows a modest positive contribution, while former smoking and NSAID use contribute minimally near the null. In contrast, several variables exhibit negative coefficients, indicating inverse associations with mortality. The strongest negative contributors are congestive heart failure and ACE inhibitor use, followed by contraindications to therapy and warfarin use. Additional modest inverse associations are observed for alcohol use and certain medication patterns. These inverse relationships likely reflect treatment selection, survivorship effects, or residual confounding rather than true protective effects. Overall, the figure demonstrates substantial heterogeneity in covariate associations in the pre-trial per-protocol setting and underscores the need for careful confounder control and cautious interpretation when estimating treatment effects from observational data.

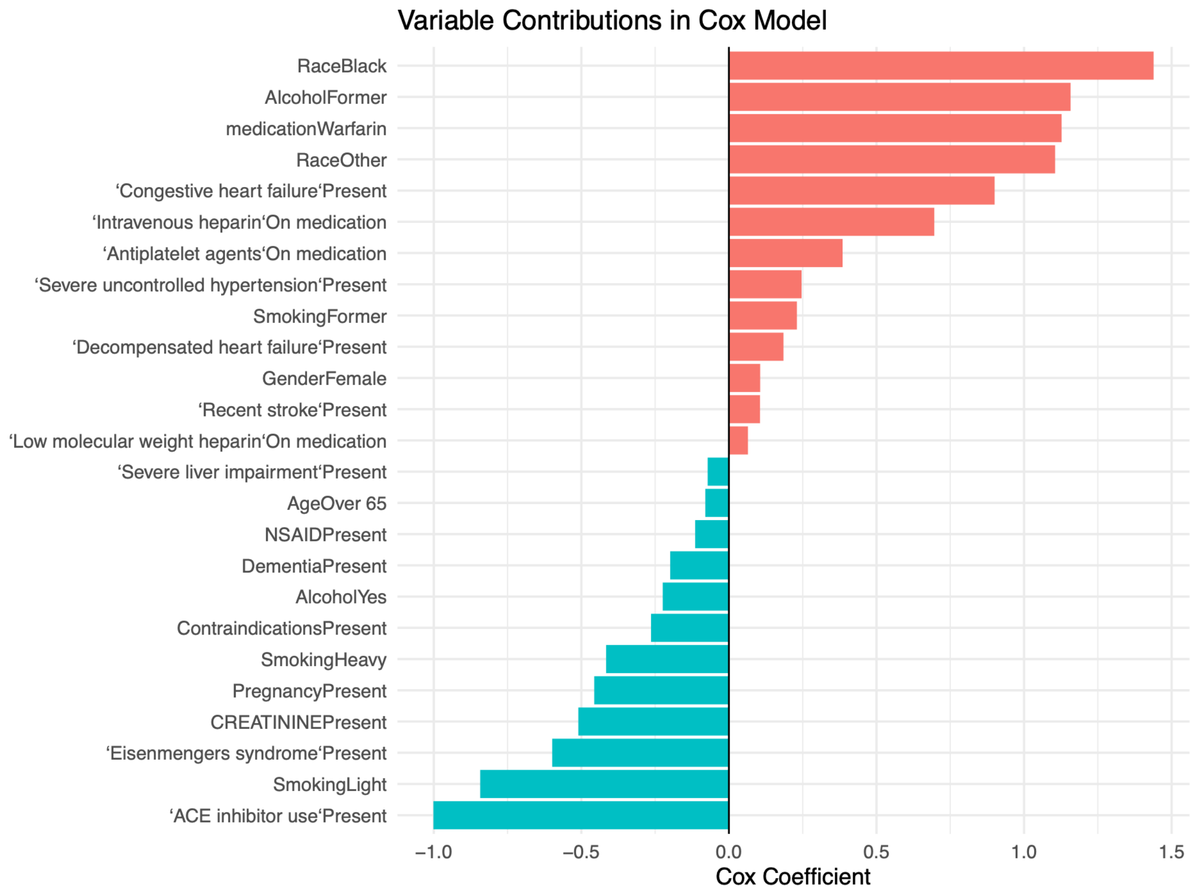


**Supplement Figure 5.** Confounders Coefficient of PP after 2014. This figure presents the most contributive clinical variables affecting the outcome in the PP analysis conducted after 2014. The horizontal bar plot ranks variables by the magnitude of their coefficients, with variable names listed on the y-axis and corresponding coefficient values on the x-axis. Positive coefficients indicate a stronger association with increased risk or higher probability of the outcome, while negative coefficients reflect a potential protective effect or reduced risk. In the post-2014 per-protocol cohort, the strongest positive associations with mortality risk are observed for Race categorized as Black, former alcohol use, and warfarin therapy, followed by Race categorized as Other, congestive heart failure, and intravenous heparin use. Additional positive contributors include antiplatelet therapy, severe uncontrolled hypertension, former smoking, decompensated heart failure, female sex, and recent stroke, indicating that demographic characteristics, cardiovascular disease burden, and treatment patterns jointly influence risk in this population. Low-molecular-weight heparin use shows a small positive contribution near the null. In contrast, several variables exhibit negative coefficients, indicating inverse associations with mortality. The largest negative effects are observed for ACE inhibitor use and light smoking, followed by Eisenmenger’s syndrome, elevated creatinine, pregnancy, heavy smoking, contraindications to therapy, alcohol use, dementia, NSAID use, and older age (≥65 years). Severe liver impairment shows only a modest inverse association. These inverse relationships likely reflect clinical selection, survivorship bias, or residual confounding rather than true protective effects. Overall, the figure demonstrates substantial heterogeneity in covariate associations after 2014 and highlights the complex interplay between demographic factors, comorbidities, and medication use in real-world per-protocol analyses.

**Supplement Table 5.** Study Summaries and Cohort Characteristics for PP Analysis.

| Characteristics | MCP Data (Before July 2014) | MCP Data (After July 2014) | Original Study Data (Overall) |
|---|---|---|---|
| Medication | 22,117 (Aspirin, 95.05%) \| 1,152 (Warfarin, 4.95%) | 4,704 (Aspirin, 95.32%) \| 231 (Warfarin, 4.68%) | N/A |
| Age | 64.95 ± 13.05 | 63.46 ± 12.85 | N/A |
| Gender | 12,616 (Male, 54.22%) \| 10,653 (Female, 45.78%) | 2,732 (Male, 55.36%) \| 2,203 (Female, 44.64%) | N/A |
| Race | 21,577 (White, 92.73%) \| 610 (Black, 2.62%) \| 687 (Other, 2.95%) \| 395 (Unknown, 1.70%) | 4,451 (White, 90.19%) \| 248 (Black, 5.03%) \| 168 (Other, 3.40%) \| 68 (Unknown, 1.38%) | N/A |
| Eisenmenger's syndrome | 372 (1.60%) | 51 (1.03%) | N/A |
| Decompensated heart failure | 3,043 (13.08%) | 502 (10.17%) | N/A |
| Heparin use | 22,928 (98.14%) | 4,880 (98.29%) | N/A |
| Dementia | 1,912 (8.22%) | 280 (5.67%) | N/A |
| Severe liver impairment | 12,476 (53.62%) | 2,929 (59.35%) | |
| Severe uncontrolled hypertension | 506 (2.17%) | 89 (1.80%) | N/A |
| Creatinine | 18,678 (80.27%) | 3,745 (75.89%) | N/A |
| ACE inhibitor use | 22,265 (95.69%) | 4,743 (96.11%) | N/A |
| Recent stroke | 943 (4.05%) | 135 (2.74%) | N/A |
| Ejection Fraction | 57.55 ± 14.71 | 56.84 ± 15.85 | N/A |
| Hypertension | 11,551 (49.44%) | 2,639 (53.15%) | N/A |
| Hyperlipidemia | 17,003 (72.78%) | 2,920 (58.81%) | N/A |
| Diabetes | 7,634 (32.68%) | 1,246 (25.10%) | N/A |
| Coronary artery disease | 12,839 (54.95%) | 1,952 (39.32%) | N/A |
